# The Influence of Physician Information on Patients’ Choice of Physician in mHealth Services Using China’s Chunyu Doctor App: Eye-Tracking and Questionnaire Study

**DOI:** 10.2196/15544

**Published:** 2019-10-23

**Authors:** Wei Shan, Ying Wang, Jing Luan, Pengfei Tang

**Affiliations:** 1 School of Economics and Management Beihang University Beijing China; 2 School of Economics and Management Beijing Jiaotong University Beijing China

**Keywords:** mHealth, physician information, choice, trust

## Abstract

**Background:**

Mobile health (mHealth) is becoming more popular as a way of sharing medical information. For the patient, it saves time, reduces the need for travel, reduces the cost of searching for information, and brings medical services “to your fingertips.” However, it also brings information overload and makes the patient’s choice of physician more difficult.

**Objective:**

This study aimed to identify the types of physician information that play a key role in patients’ choice of physician and to explore the mechanism by which this information contributes to this choice.

**Methods:**

Based on the stimulus-organism-response (SOR) model and online trust theory, we proposed a research model to explain the influence of physician information on patients’ choice of physician. The model was based on cognitive trust and affective trust and considered the moderating role of patient expertise. Study 1 was an eye-tracking experiment (n=42) to identify key factors affecting patients’ choice of physician. Study 2 was a questionnaire study (n=272); Partial Least Squares Structural Equation Modeling was used to validate the research model.

**Results:**

The results of Study 1 revealed that seven types of physician information played a key role in patients’ choice of physician. The results of Study 2 revealed that (1) physicians’ profile photo information affected patients’ choice of physician by positively influencing affective trust (*P*<.001); (2) physicians’ nonprofile photo information affected patients’ choice of physician by positively influencing cognitive trust (*P*<.001); (3) patient-generated information affected patients’ choice of physician by positively affecting cognitive trust (*P*<.001) and affective trust (*P*<.001), and patient expertise played a positive moderating role on both (*P*=.04 and *P*=.01, respectively); and (4) cognitive trust and affective trust both positively affected patients’ choice of physician, with affective trust playing a more significant role (*P*<.001 and *P*<.001, respectively).

**Conclusions:**

Seven types of physician information were mainly used by patients when choosing physicians offering mHealth services; trust played an important role in this choice. In addition, the level of patient expertise was an important variable in moderating the influence of physician information and patients’ trust. This paper supports the theoretical basis of information selection and processing by patients. These findings can help guide app developers in the construction of medical apps and in the management of physician information in order to facilitate patients’ choice of physician.

## Introduction

### Background

Information asymmetry between patients and physicians is one of the main causes of tension in the physician-patient relationship within traditional medical services [1]. Due to the lack of professional knowledge, patients are in a relatively disadvantaged position; therefore, promoting physician-patient interaction and reducing information asymmetry has been one of the focus areas of the medical industry. The term mobile health (mHealth) refers to the provision of medical information and services through mobile communication technologies and mobile devices, such as mobile phones [2]. In recent years, with the rapid development of Internet technology, the influence of mHealth in our lives has grown [3-5]. mHealth can improve the efficiency of medical services and reduce their cost through remote medical monitoring and consultation [6,7]. This new pattern of physician-patient interaction and sharing of physician information can help solve current problems in the field of medicine, such as medical inefficiency and physician-patient contradictions.

Previous research on mHealth mainly focused on its development trend [8-10]; the function, development, and design of mHealth apps [11-13]; and users’ intentions to adopt and use mHealth services [14-16]. However, in the field of mHealth user behavior, there is still a lack of in-depth research on patients’ choice of physician and its influencing factors. In fact, patients’ choice of physicians who offer mHealth is different from the choice they make within the traditional route. First, mHealth provides information about physicians far beyond the traditional medical model. On one hand, such physician information can change patients’ passive positions when accessing medical services and can improve the physician-patient relationship, further affecting their choice of physician [17-19]. On the other hand, it can also lead to information overload to some extent, which has side effects on patients; it can cause patients to feel confused when making medical choices, which could reduce their efficiency [20]. Therefore, it is important to find out what information plays a major role in patients’ choice of physician and to conduct effective information management to help patients. In addition, unlike the traditional physician-patient relationship, in the mHealth environment patients learn about physicians through mHealth platforms; they connect with each other through the network rather than via direct contact. Researchers have shown that health care is a service that requires a high amount of trust on the part of the patient [21]; in addition, trust is important for the success of online health services [22]. Therefore, patients’ trust in physicians is important for the relatively unfamiliar physician-patient relationship in mHealth. Based on the above considerations, this study focused on two important issues in mHealth: (1) the impact of physician information on patients’ choice of physician and (2) the trust mechanism between physician information and patients’ choice of physician.

To address these issues, this study focused on the Chunyu Doctor app, which is the largest mobile physician-patient communication platform in China and has attracted more than 500,000 physicians in public hospitals. Based on the stimulus-organism-response (SOR) model and online trust theory, this study explored how physician information affects patient trust in mHealth, thereby further affecting patients’ choice of physician. The role of patient expertise in the relationship between physician information and trust was also considered. Since eye-tracking methods have been used to record user behavior [23], in this study we attempted to use eye tracking to investigate the key factors that influence patients’ choice of physician. Further, a questionnaire was used to decipher the internal mechanism of this influence. This study provides a certain theoretical basis for the research of mHealth information services and provides practical guidance for the construction of mHealth apps and their information management methods.

### Theoretical Foundation

#### Online Trust

Online trust theory is based upon many studies that have classified online trust as consumer trust in e-commerce [24,25]. When consumers feel that information is insufficient or asymmetric, trust can be used as a means to alleviate the asymmetry of perceived information, prompting them to make purchasing decisions [26,27]. McAllister divided trust into cognitive trust and affective trust [28]. Cognitive trust is mainly regarded as the consumer’s rational expectation of the ability and credibility of the trusted party and is mostly judged by the objective characteristic evidence of the trusted party's personal behavior and reputation. Affective trust is regarded as a trust attitude, which reflects the consumer’s feelings and self-consciousness toward the trusted party [29-31].

Choice in mHealth can also be regarded as a special online shopping behavior. In mHealth service, if the physician is regarded as the *commodity*, then the patient is equivalent to the *consumer*. Therefore, the study of the patient’s choice in mHealth is actually the study of the consumption behavior of this particular consumer group. Asymmetry of information results in trust playing an important role in the physician-patient relationship. At present, physician-patient trust is generally defined from the patient's point of view; it is defined as the patient's trust in the physician's ability to diagnose and treat them and trust that the physician will put their interests first [32]. Therefore, patient trust plays a very important role in constructing the patient choice model. In this study, trust is also divided into cognitive trust and affective trust. In addition, according to the study by Calefato et al [33], the patient's trust in the physician's abilities (ie, physician's professional skills, knowledge, and competence) is defined as the patient's cognitive trust; the patient's trust in the physician's benevolence (ie, physician's politeness, attitude, and willingness to help) is defined as the patient's affective trust.

#### Stimulus-Organism-Response Model

The SOR model, proposed by Mehrabian and Russell in 1974, illustrates that the external environment influences the individual's attitude or behavior by influencing the individual's mental state. *Stimulus* refers to the external environmental factors received by the individual; *organism* refers to the internal mental state of the individual; and *response* refers to the attitude and behavior of the individual [34]. The model has been widely used in consumer behavior analysis. According to the model, a consumer (ie, *organism*) will have a conscious or unconscious psychological reaction after receiving an external *stimulus*, which involves both cognitive and affective aspects, and will then have an internal or external behavioral *response* to this stimulus [35-37]. In this study, physician information in the mHealth app, Chunyu Doctor, was considered an external *stimulus* that caused patients (ie, *organisms*) to generate trust, which in turn allowed them to make their choice of physicians (ie, *response*).

### Research Model and Hypotheses

#### Physician Information and Trust

Physicians are the direct providers of medical services. In the field of mHealth, physician information is one of the most important considerations for patients when choosing medical services. Patients can get detailed information about a physician when browsing the physician's home page. This study extracted physician information from two major information sources: physicians and patients. Accordingly, three kinds of information were identified: physicians’ profile photo information, physicians’ nonprofile photo information, and patient-generated information.

In the past physician-patient relationship studies, information from physicians’ profile photos was often neglected, but the face is an important source of information. Sometimes a photo can promote trust between people [38]. This face-based assessment is very subjective [39], with emotion often generating trust in others, while physicians’ nonprofile photo information is different. For example, physicians in high-level hospitals often have more advanced medical skills and richer medical experience. As well, a physician's title is a reflection of their professional skills, medical experience, and work performance. These personal traits often lead patients to trust physicians at the cognitive level.

In online trust theory, user-generated information can enhance the trust of online sellers, reduce the risk perceived by buyers, and promote transaction behavior [40]. User-generated information in mHealth corresponds to patient-generated information. The main forms of this information are comments, feedback, and scoring, which can reflect online reputation [41]. Patient-generated information is usually the first-hand experience of patients with online consultations. Eysenbach proposed that, in the field of health care, experience-based credibility can be seen as an additional dimension of source credibility [42]. In that sense, similarity between experiences will enhance patients' perceptions of credibility. In addition, some studies have suggested that patient-generated information can reveal physicians' online behavior; reduce information asymmetry between physicians and patients [43]; prevent physicians from exaggerating and misinterpreting their professional ability, service level, and treatment effect; and help patients understand physicians' medical expertise and service attitude [44]. In conclusion, patient-generated information will affect patients' trust in physicians at the cognitive and affective levels, thus affecting their behavior in choosing physicians. Therefore, the following hypotheses are put forward:

Hypothesis 1a (H1a): A physician’s nonprofile photo information will positively affect the patient's cognitive trust.

Hypothesis 1b (H1b): A physician’s profile photo information will positively affect the patient's affective trust.

Hypothesis 1c (H1c): Patient-generated information will positively affect the patient's cognitive trust.

Hypothesis 1d (H1d): Patient-generated information will positively affect the patient's affective trust.

#### Patient Expertise, Physician Information, and Trust

Consumer expertise is a key factor influencing a consumer’s choice; it is the knowledge and experience, of which a consumer is subjectively aware, regarding a particular product or service [45]. High-expertise consumers are willing to take the initiative to look for information related to products and to make decisions through careful thinking because of their rich experience. Low-expertise consumers are more passive in searching for information, preferring to rely on marginal information, and are less willing to spend time thinking about new information.

In consumer behavior research, consumer expertise plays a significant role in explaining consumer decisions and responses to products [46,47]. In this study, patient expertise refers to the patient’s knowledge or experience of mHealth services. Since patient-generated information is unique information in mHealth compared with traditional medical services, we believe that patient expertise will play a moderating role between physician information and trust. That is, the higher the expertise of patients, the more they will rely on patient-generated information in choosing a physician; however, low-expertise patients will rely more on the physician’s profile photo and nonprofile photo information. Hence, we hypothesize the following:

Hypothesis 2a (H2a): Patient expertise will play a negative role in the influence of a physician’s nonprofile photo information on cognitive trust.

Hypothesis 2b (H2b): Patient expertise will play a negative role in the influence of a physician’s profile photo information on affective trust.

Hypothesis 2c (H2c): Patient expertise will play a positive role in the impact of patient-generated information on cognitive trust.

Hypothesis 2d (H2d): Patient expertise will play a positive role in the impact of patient-generated information on affective trust.

#### Trust and Choice of Physician

Corritore and Wiedenbeck [22] proposed that in online medical care, physician-patient trust is one of the most important factors to ensure the success of online health services. Gefen et al [48] believed that consumers’ intentions to use certain goods or services mainly depended on consumers' cognitive trust and affective trust in external factors. Therefore, in this study, patients’ trust is considered to influence patients’ choice of physician in mHealth. Hence, we hypothesize the following:

Hypothesis 3a (H3a): The patient's cognitive trust in a physician will positively influence his or her choice of physician.

Hypothesis 3b (H3b): The patient's affective trust in a physician will positively influence his or her choice of physician.

**Figure 1:**
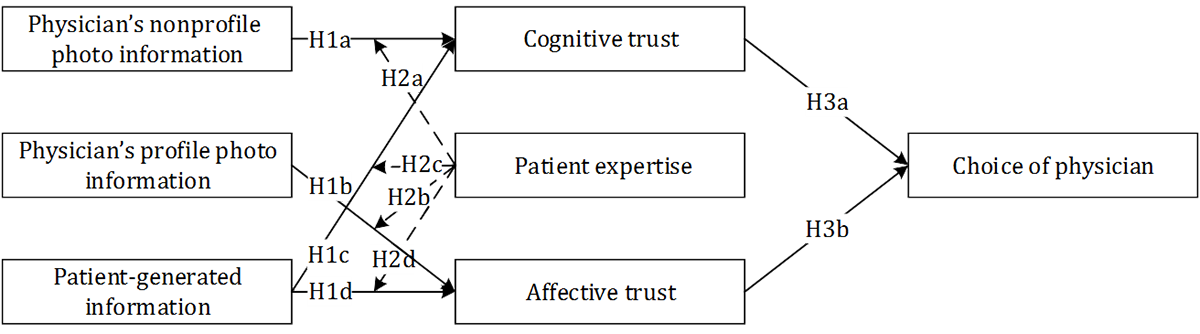
Research model explaining the influence of physician information on the patients’ choice of physician in mHealth. H1a: hypothesis 1a; H1b: hypothesis 1b; H1c: hypothesis 1c; H1d: hypothesis 1d; H2a: hypothesis 2a; H2b: hypothesis 2b; H2c: hypothesis 2c; H2d: hypothesis 2d; H3a: hypothesis 3a; H3b: hypothesis 3b.

Based on our hypotheses, this study illustrates a research model that explains the influence of physician information on the patients’ choice of physician in mHealth, as shown in Figure 1.

## Methods

### Overview

Two studies were conducted. Study 1 aimed to identify the key physician information that had an impact on patients’ choice of physician in mHealth through an eye-tracking experiment. Study 2 used questionnaires to explore what influence physician information, determined in Study 1, had on patients’ choice of physician.

### Study 1: Investigating Physician Information

#### Research Design

The purpose of this study was to investigate the impact that physician information had on patients’ choice of physician in an mHealth platform and to identify the information that played a key role in this choice. Research has shown that people's ability to process information within their short-term memory is limited [49]; therefore, even though the information provided in the mHealth app, Chunyu Doctor, is comprehensive, a patient’s choice of physician is based only on a limited amount of useful information. In previous online medical studies, scholars have often used their own experiences and related theories to select the information they think is more useful, built regression models by crawling data to uncover the relationship between the physician information and the number of physicians’ consultations, and judged the impact of physician information on patients’ choice of physician [50-52]. This method is subjective and it is easy to overlook some potentially critical information. Humans obtain much external information through their vision and the images to which their attention is focused [53]. Through eye-tracking experiments, researchers can capture the information upon which people focus their attention more objectively and accurately. Therefore, in this study, eye-tracking experiments were used to screen physician information that had a key impact on patients’ choice of physician.

The Chunyu Doctor app contains 12 different types of physician information; for the universality of the experimental results, we did not consider the physicians' departments or specific communication between physicians and patients. The types of information we were concerned with were divided into three categories: physicians’ profile photo information, physicians’ nonprofile photo information, and patient-generated information. This information is shown in Table 1.

In this study, the 12 types of physician information (see Table 1) were classified into high-level and low-level information. Hospital, title, and educational background levels were classified by the government (eg, hospitals are classified into Class A tertiary hospitals and general hospitals); because of this, other information levels, except physicians’ profile photo information, were set with reference to the Chunyu Doctor app and all of them were pretested using a questionnaire. The questionnaire showed that the various information levels were significantly different; the specific classification is shown in Multimedia Appendix 1.

**Table 1:** Classification of physician information.

Information category	Types of information
Physicians’ profile photo information	Profile photo
Physicians’ nonprofile photo information	Hospital, title, educational background, academic research results, topic, fees, and peer evaluations
Patient-generated information	Consultation numbers, favorability rate, satisfaction, and gratitude expressed

Regarding physicians’ profile photo information, based on a study by Ert et al [54], two dimensions were used to distinguish the information level: attractiveness and trustworthiness. A total of 60 participants—28 males (47%) and 32 females (53%)—rated 40 physician profile photos based on their perceived attractiveness and trustworthiness. We used photos of physicians from the official website of Peking Union Medical College Hospital; 20 males and 20 females, aged 30-40 years old, were selected. In the photos, all physicians wore glasses, white coats, and light sweaters or shirts; none had beards, accessories, or visible makeup. All photos were front-facing portraits of physicians smiling. Each participant had to look at all 40 physician profile photos. For each physician's profile photo, participants were asked to answer the following questions: “Do you think the physician is attractive?” and “Do you think the physician is trustworthy?” These were asked in order to estimate the perceived levels of attractiveness and trustworthiness of the profile photos. The questions were scored on a 7-item Likert scale, ranging from 1 (*totally unattractive/untrustworthy*) to 7 (*very attractive/trustworthy*); the order in which the photos appeared to participants was random. Eight profile photos were selected based on the photos’ average attractiveness and trustworthiness levels; four photos had the highest and four photos had the lowest average attractiveness and trustworthiness levels, respectively. There was a significant difference between high-level and low-level profile photos (*F*_1_=94.58, *P*<.001); however, there was no significant difference between the four high-level profile photos (*F*_3_=0.622, *P*=.60) and the four low-level profile photos (*F*=0.292, *P*=.83). In addition, there was no significant difference between male and female physicians (*F*_1_=2.547, *P*=.11).

In this study, 12 types of physician information were grouped (see Table 1) and divided into three major categories. Each category was classified according to the rated levels (eg, high-level patient-generated information contained high consultation numbers, high praise rates, high satisfaction, and high gratitude expressed; low-level patient-generated information contained low consultation numbers, low praise rates, low satisfaction, and low gratitude expressed). This was used to generate eight mHealth physician home pages. In summary, the eye-tracking experiment was a 2 (physicians’ profile photo information: high or low level) × 2 (physicians’ nonprofile photo information: high or low level) × 2 (patient-generated information: high or low level) within-group experiment, in which eye-tracking data and questionnaire data were collected.

#### Procedure

A total of 42 undergraduate and postgraduate students from Beihang University participated in the experiment; participants were publicly recruited via the Chinese social media platform WeChat. In order to avoid gender bias, 21 men (50%) and 21 women (50%), aged 20-25 years old, were recruited. All participants had normal visual acuity or corrected visual acuity, with astigmatism below 200 degrees, and had online shopping experience. Before beginning the experiment, each participant signed an informed consent letter and registered their personal information. After the experiment, participants were compensated with CNY ¥30.

The eye-tracking equipment used in this study was the Tobii T120 eye tracker (Tobii Technology). The eye tracker was integrated into a 17-inch, thin-film-transistor display with a resolution of 1280 × 1024 and a sampling rate of 120 Hz. The instrument has a built-in eye-tracking server; the participants did not need to wear any equipment but did need to watch the experimental material displayed on the monitor. While the participants looked at the screen, the eye tracker automatically recorded eye-tracking data of the participants. Eye-tracking data can be analyzed and extracted using the Tobii Studio software associated with the eye tracker.

In order to simulate the mHealth treatment situation as realistically as possible, the experimental interface was based on the physician’s home page on the Chunyu Doctor app. In order to avoid interference from other information, only the aforementioned 12 types of physician information were included. Each physician's home page was the same size and, as much as possible, the display format of the various types of information was consistent with the original app. The experiment began with an introduction:

We assume that you or a family member has a chronic disease, such as a sports injury, psychological stress, chronic disease, cold, fever, physical discomfort, etc, and you want to consult with your physician about the relevant information and treatment of the disease. In this experiment, you will be shown information about eight different physicians. These physicians are on the Chunyu Doctor mHealth app and work in a department related to the disease about which you want a consultation. After reading information about each physician, you will need to rate the statement “I choose this physician.” The options range from 1 (strongly disagree) to 7 (strongly agree). Please complete the questionnaire according to your subjective feelings.

During the introduction to the experiment, two unfamiliar types of information were explained, namely, gratitude expressed (ie, offering extra thank-you tips) and physician's topic (ie, number of physician's articles published). Participants were able to view the experimental stimuli (ie, the Chunyu Doctor app) by clicking the left mouse button on the screen. In order to ensure that the participants were able to fully observe the information about the eight physicians, each physician's home page was fixed for 1 minute. The eye-tracking experiment was complete after participants browsed the home pages of the eight physicians. Participants were then asked to complete a questionnaire; participants needed to rate the usefulness of the 12 types of information by rating the following statement for each on a scale from 1 (*strongly disagree*) to 7 (*strongly agree*): “I think this information is very useful for me in helping me choose this physician.” At this point, the experiment has been completed.

### Study 2: The Mechanism of Influence of Physician Information on Patients’ Choice

Study 2 was based on the physician information identified from Study 1 that played a key role in patients’ choice. In this part of the study, we investigated how physician information affected patients' choice of physician. Questionnaires were used to collect sample data through a 2 (physician’s profile photo information: high or low level) × 2 (physician’s nonprofile photo information: high or low level) × 2 (patient-generated information: high or low level), between-group experiment to verify our research model. We combined the three categories of physician information to generate home pages for each of the eight physicians. Only the information identified from Study 1 that played a key role in patients’ choice appeared on the home pages in Study 2. However, because fees are often related to physicians’ hospitals and titles, the impact of fees on trust was not considered in this study. Each participant only saw one of the eight physician home pages, which was chosen at random. The questionnaire was divided into three parts: (1) collection of demographic information (eg, age, gender, educational level, online shopping experience, and frequency of use of mHealth apps); (2) measurement of the moderator variable, patient expertise; and (3) participants’ responses regarding cognitive trust, affective trust, and physician choice after observing each physician’s home page. Each variable was measured using a 7-item Likert scale, ranging from 1 (*strongly disagree*) to 7 (*strongly agree*). The design of the questionnaire drew on the relevant literature; appropriate adjustments were made according to the content of this study to ensure the validity and reliability of the constructed measures, as shown in Table 2. Questionnaires were distributed through the Questionnaire Star platform (China Wise Talent Information Technology Co), a professional online questionnaire survey service in China.

**Table 2:** Constructs and corresponding items.

Construct	Items
Cognitive trust (CT) [55]	CT1: This physician was competent and effective in meeting my needs. CT2: This physician was capable and proficient. CT3: This physician was very knowledgeable in his or her medical field.
Affective trust (AT) [33,55]	AT1: This physician would act in my best interest. AT2: If I required help, this physician would do his or her best to help me. AT3: I think this physician is friendly and approachable.
Choice of physician (CP) [56]	CP1: I would be willing to choose this physician. CP2: I would be willing to recommend this physician to others. CP3: I have positive things to say about this physician.
Patient expertise (PE) [46]	PE1: I am knowledgeable about mHealth services. PE2: I learn well about mHealth services. PE3: I have rich experience in mHealth services.

## Results

### Study 1: Investigation of Physician Information

**Figure 2:**
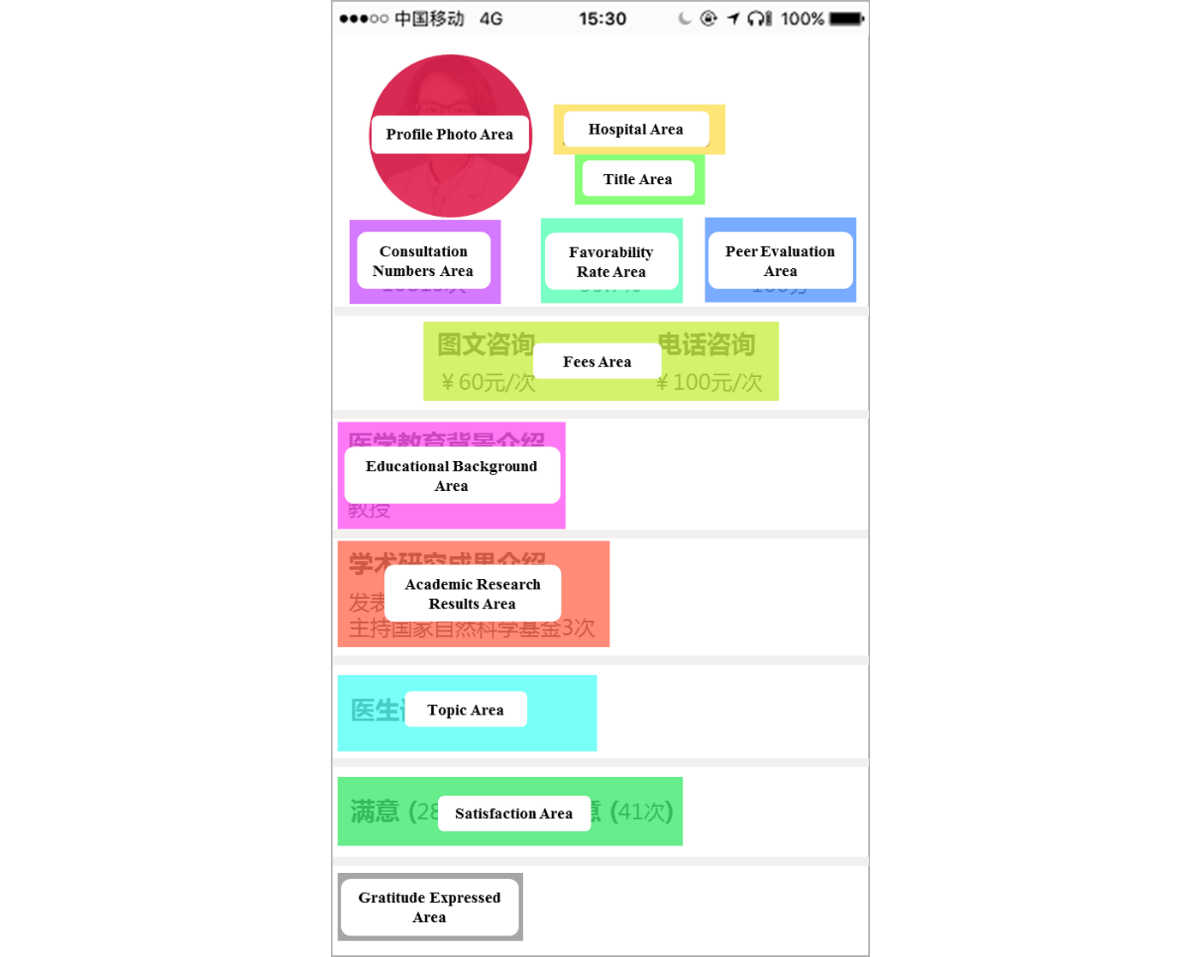
Area of interest division map of physician home pages.

The area of interest (AOI) in eye-tracking experiments is the gaze area to which researchers pay attention and represents the stimulus information in which participants are interested. AOI is the basic unit of analysis in eye-tracking experiment results. According to the classification of physician information as shown in Figure 2, the interface of each physician's home page was divided into 12 corresponding AOIs: profile photo, hospital, title, consultation numbers, favorability rate, peer evaluation, fees, educational background, academic research results, topic, satisfaction, and gratitude expressed. Based on previous literature, the average duration of visual fixation for each AOI was selected as the eye-tracking analysis index for this experiment. The average duration of fixation is the average time in seconds of fixation per unit area in the AOI. The longer the average duration of fixation, the more interested the participants were in the test material or the more difficult it was for them to extract information [57]. In order to identify the information that participants were more interested in rather than information that was difficult to extract based on the eye-tracking index, it was necessary to combine the results of the questionnaire. John Miller, an American psychologist, has accurately measured the capacity of short-term memory; this capacity for a normal adult is 7 ± 2 items at a time, with memory ability decreasing with more than seven items [49]. Therefore, this study used the average ranking of eye-tracking data (ie, average duration of fixation) and questionnaire data (ie, ranking of the usefulness of information) to ultimately select seven aspects of information that had a key impact on patients’ choice of physician: favorability rate, consultation numbers, title, hospital, satisfaction, profile photo, and fees. The specific data are shown in Table 3.

As the results show in Table 3, we observed an interesting finding. People often subjectively believe that patient-generated information (ie, favorability rate, satisfaction, consultation numbers, and gratitude expressed) is more useful to them in choosing a physician than a physician’s profile photo. This may also be the reason why physicians’ profile photo information has not been taken into account in previous studies. However, eye-tracking data revealed contradictory results; according to this data, people payed much more attention to the profile photos. It can be seen that a physician’s profile photo is also a very important factor in patients’ choice of physician.

**Table 3:** Ranking of 12 types of physician information.

Item #	Physician information	Duration of fixation (seconds), mean (SD)	Rank for duration of fixation	Information usefulness^a^	Rank for information usefulness	Ranking average
1	Favorability rate	8.14 (3.45)	2	5.88	1	1.5
2	Consultation numbers	7.22 (2.39)	4	5.74	3	3.5
3	Title	8.30 (4.55)	1	4.89	6	3.5
4	Hospital	6.74 (5.68)	5	5.08	5	5.0
5	Satisfaction	4.71 (2.31)	9	5.85	2	5.5
6	Profile photo	7.30 (3.96)	3	4.22	9	6.0
7	Fees	5.80 (1.72)	6	4.76	7	6.5
8	Gratitude expressed	4.50 (2.58)	10	5.58	4	7.0
9	Educational background	4.77 (1.28)	8	4.39	8	8.0
10	Peer evaluations	5.03 (2.16)	7	3.60	12	9.5
11	Academic research results	4.09 (1.25)	11	4.22	10	10.5
12	Topic	3.84 (1.55)	12	3.88	11	11.5

^a^Participants rated the usefulness of the 12 types of information by rating the following statement for each on a scale from 1 (*strongly disagree*) to 7 (*strongly agree*): “I think this information is very useful for me in helping me choose this physician.”

### Study 2: The Mechanism of Influence of Physician Information on Patients’ Choice of Physician

#### Sample Characteristics

A total of 272 questionnaires were recovered, of which 254 (93.4%) were valid. The sample distribution characteristics are shown in Multimedia Appendix 2. Among the participants, 44.5% (113/254) were male and 55.5% (141/254) were female. A total of 87.4% (222/254) of the participants were under 30 years old and all of them had online shopping experience. In line with the fact that young people are more receptive to new things, mobile device users tend to be younger and older people mostly use mHealth apps via their children. A total of 42.1% (107/254) had never used mHealth services while 57.9% (147/254) had used them; 17.7% (45/254) had used them one or more times a week. These findings could help explain the patient expertise in mHealth of the study participants. Based on the above analysis, the study sampling was considered reasonable.

#### Reliability and Validity

The reliability and validity of the measurement instruments were tested [58,59], as shown in Tables 4 and 5. SPSS, version 22.0 (IBM Corp), was used to analyze the reliability of the data and to measure the Cronbach alpha values of each construct. The principal component analysis showed that the Kaiser-Meyer-Olkin value of the sample was 0.904, which indicated that it was suitable for factor analysis. SmartPLS 3.0 (SmartPLS GmbH) was used to carry out confirmatory factor experiments [60] to analyze the validity of the data, including convergence validity and discriminant validity. Table 4 shows that the Cronbach alpha values of all constructs were greater than .8 and that the composite reliability values were also greater than 0.8, which shows that the measured constructs had better reliability. The factor loads of all variables were greater than 0.7. The average variance extraction (AVE) values were greater than 0.5, which indicated that the constructs in this study had good convergence validity [61].

For the test of discriminant validity of the measurement scale, we can compare the square root of each factor’s AVE and its correlation coefficients with other factors [61]. The results of the discriminant validity measurement are shown in Table 5, in which the enlarged diagonal values are the square root of each factor’s AVE. Table 5 shows that the square root of each factor’s AVE is greater than the corresponding correlation coefficients, which indicates that the discriminant validity between the constructs in this study was quite good.

**Table 4:** Construct reliability and convergence validity.

Construct amd items		Factor load	Cronbach alpha	Composite reliability	Average variance extraction
**Cognitive trust (CT)**			.950	0.968	0.909
	CT1: This physician was competent and effective in meeting my needs.	0.938			
	CT2: This physician was capable and proficient.	0.967			
	CT3: This physician was very knowledgeable in his or her medical field.	0.955			
**Affective trust (AT)**			.903	0.940	0.838
	AT1: This physician would act in my best interest.	0.885			
	AT2: If I required help, this physician would do his or her best to help me.	0.937			
	AT3: I think this physician is friendly and approachable.	0.924			
**Choice of physician (CP)**			.899	0.937	0.832
	CP1: I would be willing to choose this physician.	0.932			
	CP2: I would be willing to recommend this physician to others.	0.932			
	CP3: I have positive things to say about this physician.	0.872			
**Patient expertise (PE)**			.906	0.941	0.841
	PE1: I am knowledgeable about mHealth services.	0.916			
	PE2: I learn well about mHealth services.	0.921			
	PE3: I have rich experience in mHealth services.	0.914			

**Table 5:** Discriminant validity analysis.

Construct	Cognitive trust	Affective trust	Choice of physician	Patient expertise
Cognitive trust	0.953			
Affective trust	0.748	0.916		
Choice of physician	0.812	0.829	0.912	
Patient expertise	0.155	0.242	0.228	0.917

#### Analysis of Research Model

Partial Least Squares Structural Equation Modeling (PLS-SEM) has advantages over Covariance-Based Structural Equation Modeling (CB-SEM) or general linear regression modeling in processing sample data that do not obey normal distribution—physician information is a class variable—involving multiple constructs and small sample size prediction experiments [62]. Therefore, we used SmartPLS 3.0, an analysis software commonly used in partial least squares path modeling, to construct the whole structural equation; we used bootstrapping with 5000 resamples to assess the statistical significance of path coefficients [63].

The results show that 77.1% of the variation in patient choice could be explained by the model (R^2^=0.771). Table 6 shows that most of the hypotheses in this model were supported. Physicians’ nonprofile photo information had a significant positive impact on cognitive trust. Physicians’ profile photo information had a significant positive impact on affective trust, which supports H1a and H1b. Patient-generated information had a significant positive impact on cognitive trust and affective trust, which supports H1c and H1d. Cognitive trust and affective trust had significant positive effects on patients’ choice of physician, which supports H3a and H3b. However, the hypotheses regarding the moderating effect of patient expertise were only partially supported. Patient expertise had significant moderating effects on the influence of patient-generated information, both on cognitive trust and affective trust. That is, when patient expertise was higher, the level of information generated by patients was higher and they experienced more cognitive and affective trust; thus, H2c and H2d were supported. However, patient expertise had no significant moderating effect on the influence of physicians’ nonprofile photo information on cognitive trust nor on physicians’ profile photo information on affective trust. Therefore, H2a and H2b were not supported.

**Figure 3:**
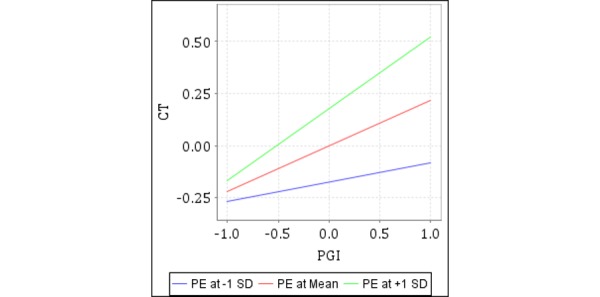
Moderating effect of patient expertise (PE) on cognitive trust (CT) by patient-generated information (PGI).

**Figure 4:**
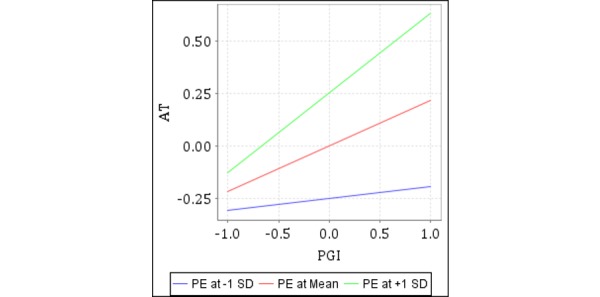
Moderating effect of patient expertise (PE) on affective trust (AT) by patient-generated information (PGI).

To sum up, this study showed that in mHealth, physician information affected patients’ choice of a physician through trust. In addition, it should be noted that patients with different levels of expertise will have different experiences. The experimental results showed that patient expertise had no significant influence on the relationship between physicians’ profile photo information and affective trust nor between physicians’ nonprofile photo information and cognitive trust. It may be because physicians’ profile photo information and physicians’ nonprofile photo information are widely known types of information that exist in online medical treatment; no matter how much knowledge and experience patients have regarding mHealth care, it will not change the impact that these two types of information have on patients' choice of physician. However, patient expertise had a significant impact on the relationship between patient-generated information and cognitive trust and on the relationship between patient-generated information and affective trust. The interaction diagrams of the moderating effects are shown in Figures 3 and 4.

**Table 6:** Results of hypothesis testing.

Hypothesis	Path	Path coefficient	*t* test	*P* value	Supported?
H1a: A physician’s nonprofile photo information will positively affect the patient's cognitive trust.	PNI^a^→CT^b^	0.258	4.558	<.001	Yes
H1b: A physician’s profile photo information will positively affect the patient's affective trust.	PPI^c^→AT^d^	0.174	3.197	.001	Yes
H1c: Patient-generated information will positively affect the patient's cognitive trust.	PGI^e^→CT	0.218	3.764	<.001	Yes
H1d: Patient-generated information will positively affect the patient's affective trust.	PGI→AT	0.217	3.759	<.001	Yes
H2a: Patient expertise will play a negative role in the influence of a physician’s nonprofile photo information on cognitive trust.	PNI × PE^f^→CT	0.097	0.933	.17	No
H2b: Patient expertise will play a negative role in the influence of a physician’s profile photo information on affective trust.	PPI × PE→AT	–0.068	1.013	.16	No
H2c: Patient expertise will play a positive role in the impact of patient-generated information on cognitive trust.	PGI × PE→CT	0.127	1.730	.04	Yes
H2d: Patient expertise will play a positive role in the impact of patient-generated information on affective trust.	PGI × PE→AT	0.161	2.251	.01	Yes
H3a: The patient's cognitive trust in a physician will positively influence his or her choice of physician.	CT→CP^g^	0.437	7.553	<.001	Yes
H3b: The patient's affective trust in a physician will positively influence his or her choice of physician.	AT→CP	0.502	8.696	<.001	Yes

^a^PNI: physicians’ nonprofile photo information.

^b^CT: cognitive trust.

^c^PPI: physicians’ profile photo information.

^d^AT: affective trust.

^e^PGI: patient-generated information.

^f^PE: patient expertise.

^g^CP: choice of physician.

## Discussion

### Principal Findings

Based on the SOR conceptual framework, this paper explored the impact of different types of physician information on an mHealth app on patients’ choice of physician. In the era of Network 2.0, a large amount of data will be delivered to consumers at little cost; with it comes cognitive load, so consumers are often willing to take shortcuts when making assessments or decisions [64]. Therefore, we believe that, although there is an increasing amount of physician information on mHealth apps, this is part of the information that plays a key role in patients’ choice of physician. Such information—physicians’ nonprofile photo information, physicians’ profile photo information, and patient-generated information—influences patients’ choice of physician through cognitive trust and affective trust and is regulated by patient expertise. In order to support our hypotheses, two studies were designed.

First, based on the capacity of human short-term memory, in Study 1 we discovered seven aspects of physician information that play a key role in patients’ choice of physician through an eye-tracking experiment: physicians’ profile photo, hospital, title, favorability rate, consultation numbers, satisfaction, and fees. Through this, we discovered an interesting phenomenon. Most of the participants subjectively believed that physicians’ profile photos were not very useful when making their choices; however, our preliminary evidence showed that the influence of the visual information from the physicians’ profile photos was beyond what they believed. In the field of online medical treatment, the influence of physicians’ profile photos on patients’ choice of physician has not been systematically discussed. Previous studies have suggested that smiling may increase consumers' perceived trust, and the attractiveness and perceived trustworthiness of profile photos may be related to each other, leading to the *beauty premium* phenomenon [54,65,66]. Based on this, we believe that physicians’ profile photos are an influential factor that play a very important role in patients’ choice of physician in mHealth services; this may create some potential economic benefits. Therefore, future research on visual information should be expanded.

Second, in Study 2 we found that physicians’ profile photo information and physicians’ nonprofile photo information positively influenced patients’ choice of physician through affective trust and cognitive trust, respectively, which was not surprising. However, patient-generated information positively affected patients’ choice of physician through cognitive trust and affective trust; patient expertise played a significant moderating role, that is, the higher the expertise of patients, the greater the role of patient-generated information. mHealth services include a process of interaction between physicians and patients. When patients make a choice, they not only pay attention to physicians’ personal information, but are also influenced by other patients' suggestions to a large extent. Previous studies investigated the impact of user-generated information but generally concentrated on books, movies, and other consumer goods [67,68]; there is little literature on medical services. Intangible, heterogeneous service quality is often more difficult to evaluate than product quality [69]; this study fills the research gap in the medical field. Moreover, we believe that with the continuing development of the Internet, mHealth care will be further popularized and people's patient expertise regarding mHealth care will be improved; this means that patient-generated information will play a greater role.

We discovered another interesting phenomenon from our study results; affective trust played a more important role on patients’ choice of physician than did cognitive trust. This supports the theory proposed by Komiak and Benbasat that in the field of e-commerce, affective trust plays a more important role than cognitive trust in determining consumers' willingness to adopt new services [70]. This discovery could be positive for some junior physicians. Nowadays, the competition for talent is becoming more fierce; some physicians are often unable to secure employment in higher-quality hospitals or they fail to receive higher-ranked titles because of their relatively fewer qualifications. Although these may result in lower cognitive trust from patients, these physicians can make efforts in the direction of affective trust. This can include actively improving their personal photos by looking more formal and sporting professional smiles to increase their visual credibility. In addition, physicians can devote more energy to mHealth services, treat each patient with the utmost care and professionalism, and allow patients to benefit from their goodwill in order to improve patient-generated information, which could help improve their competitiveness in mHealth services.

### Implications

The results of this study may also contribute to the development of mHealth apps; mHealth apps can be designed and improved upon in the following three aspects.

First, the patients’ eye-tracking behavior on the mHealth platform and questionnaire results can be combined to determine the most efficient and effective way to sort information within apps. Then, information can be arranged according to the phenomenon that, in eye-tracking experiments, a user's attention has been shown to decrease from top to bottom and from left to right. According to this study, patients paid the most attention to favorability rate and the consultation numbers, so information such as this can be placed at the top left.

Second, mHealth platforms should actively improve the relevant mechanism of patient-generated information, considering its influence on patient trust; this would allow it to impact and promote the successful establishment of physician-patient relationships.

Third, mHealth platforms can authenticate patient expertise by setting up questionnaires. When setting up a physician recommendation list, platforms should consider giving more weight to patient-generated information (eg, favorability rate, consultation numbers, and satisfaction) when patient expertise is high. When patient expertise is low, platforms should consider giving more weight to physicians’ profile photo information as well as their hospital and title information.

### Limitations

The results from this study help in understanding the process of information selection, the usage of mHealth apps, and information that influences patients’ choice regarding medical services. However, there are still some limitations that will need be addressed in future research. First, only 12 types of physician information were selected in this study; however, other information, such as patient comments and physician-patient conversation records, may also have an impact on patients’ choice of physician. Future research can address the impact of these and other types of information. Second, mHealth care could play a relatively prominent role among the elderly [16], however, the participants of this study were younger (ie, 20-25 years of age in Study 1 and mainly under 30 years of age in Study 2). Follow-up research could include participants in other age groups in order to promote the widespread use of mHealth apps. In addition, the questionnaire survey method used in this study had certain subjective limitations. Follow-up research could apply the in-depth case study method, as it has advantages over the questionnaire method when analyzing dynamic phenomena [71].

## Appendix

Multimedia Appendix 1 Physician information levels.

Multimedia Appendix 2 Demographic characteristics of the participants.

## Abbreviations

AOI: area of interest
AT: affective trust
AVE: average variance extraction
CB-SEM: Covariance-Based Structural Equation Modeling
CP: choice of physician
CT: cognitive trust
H: hypothesis
mHealth: mobile health
PE: patient expertise
PGI: patient-generated information
PLS-SEM: Partial Least Squares Structural Equation Modeling
PNI: physicians’ nonprofile photo information
PPI: physicians’ profile photo information
SOR: stimulus-organism-response
