# Supplementary material for: The Influence of Physician Information on Patients’ Choice of Physician in mHealth Services Using China’s Chunyu Doctor App: Eye-Tracking and Questionnaire Study
Source: JMIR Mhealth Uhealth. 2019 Oct 23;7(10):e15544. doi: 10.2196/15544 (PMC6913723; doi:10.2196/15544)
Supplement: Multimedia Appendix 1 [file mhealth_v7i10e15544_app1.pdf]

Table A. Physician Information Level

Physician information	High level	Low level
Hospital	Class A tertiary hospital	General hospital
Title	Chief physician	Physician
Educational background	Doctor of Medicine, Professor	Bachelor degree
Academic research results	Published 20 papers and presided 3 projects of the National Natural Science Foundation of China	No specific academic research results
Topic	10 topics	0 topic
Fees	CNY ¥60 for graphic consultation, CNY ¥100 for telephone consultation	CNY ¥60 for graphic consultation, CNY ¥100 for telephone consultation
Peer evaluations	100 points	98 points
Consultation numbers	10815 times	623 times
Favorability rate	99.7%	90.3%
Satisfaction	Satisfaction (2857 times), unsatisfactory (41 times)	Satisfaction (83 times), unsatisfactory (10 times)
Gratitude expressed	552 times	3 times
