# Supplementary material for: The Influence of Physician Information on Patients’ Choice of Physician in mHealth Services Using China’s Chunyu Doctor App: Eye-Tracking and Questionnaire Study
Source: JMIR Mhealth Uhealth. 2019 Oct 23;7(10):e15544. doi: 10.2196/15544 (PMC6913723; doi:10.2196/15544)
Supplement: Multimedia Appendix 2 [file mhealth_v7i10e15544_app2.pdf]

Table B. Demographic characteristics of the participants (N=254)

Characteristic	Number of participants, n (%)
**Gender**	
Male	113 (44.5)
Female	141 (55.5)
**Age**	
20	7 (2.8)
20-30	215 (84.6)
30-40	18 (7.1)
40	14 (5.5)
**Education level**	
High school and below	11 (4.3)
Specialist college	6 (2.4)
Undergraduate degree	84 (33.1)
Master's degree and higher	153 (60.2)
**Online Shopping Age**	
0 year	0 (0)
1-5 years	85 (33.5)
5-10 years	141 (55.5)
Over 10 years	28 (11)
**Frequency of using mHealth service**	
Once or more a week	45 (17.7)
Once a month	25 (9.8)
Once in two months or even longer	77 (30.3)
Never	107 (42.1)
